# Spatiotemporal Evolution and Factors Influencing Ecological Civilization Development in Chinese Watersheds

**DOI:** 10.3390/ijerph191710728

**Published:** 2022-08-29

**Authors:** Binsen Chen, Bin Zhao, Yi Li, Qiuyue Yu, Bingjian Zhao, Junyin Tan, Chuanhao Wen

**Affiliations:** 1 Research Center for Economy of Upper Reaches of the Yangtze River, Chongqing Technology and Business University, Chongqing 400067, China; 2 School of Economics, Xihua University, Chengdu 610039, China; 3 School of Economics, Lanzhou University, Lanzhou 730000, China; 4 School of Economics, Yunnan University, Kunming 650091, China

**Keywords:** ecological civilization, watershed, factor analysis method

## Abstract

The construction of ecological civilization plays an important role in realizing the harmonious coexistence between man and nature. The aims of this study were to explore the development of ecological civilization in China’s top 10 river basins from 2004 to 2018 and construct an evaluation index system of ecological civilization. Factor analysis was used for the evaluation, and intergroup gap and panel regression analyses were utilized to determine the evolution of the spatiotemporal patterns and factors affecting the development level of ecological civilization in Chinese river basins. The results show that areas with a high level of ecological civilization development gradually spread to peripheral basins such as the Liaohe, Yellow, and Songhua River basins. The level of ecological civilization in China’s watersheds is undergoing continuous development. The degree of opening up, forest cover, and education have markedly positive effects on the development of ecological civilization in the basins, whereas urban development and financial autonomy have significant negative effects. The results of this study provide new ideas for evaluating the level of ecological civilization construction, as well as a reference for the government to formulate policies related to the construction of ecological civilization in river basins.

## 1. Introduction

On 5 June 1972, the first International Conference on Environmental Protection-United Nations Conference on Human Environment was held in Stockholm, Sweden. In total, 1300 delegates from 113 countries attended the conference, representing the first gathering of governments around the world to discuss contemporary environmental issues and global environmental protection strategies [1]. To reexamine serious problems of the Earth’s environment and economic development and conceive realistic solutions, humans have carried out extensive and in-depth exploration since the Stockholm Conference. In recent years, the Chinese government has put forward the idea of constructing the world’s first ecological civilization, which has become another major exploration to solve the contradiction between environmental protection and economic development. Based on the construction of ecological civilization, the Chinese government has made important achievements in environmental protection while achieving economic growth [2]. However, a series of questions remain to be answered: How can the development level of ecological civilization be scientifically and reasonably evaluated? What are the factors affecting the development of ecological civilization? How can ecological civilization be better built? 

Academic circles have carried out research regarding ecological civilization. First, they needed to reach a consensus on the concept and connotation of ecological civilization. The concept of “ecological civilization” was first promoted by Hu Jintao during the 17th National Congress of the Chinese Communist Party in 2007 [3]. It became an integral part of the 2011, 12th Five Years Plan and in 2013, during the third plenary session of the 18th Party Congress, it was included as one of the five national objectives (socialist economical construction, political construction, cultural construction, social construction, and ecological civilization construction) [3]. Ecological civilization is defined as a Chinese adaption of the sustainable development concept [4]. Second, several scholars have adopted an analytic hierarchy process, grey correlation analysis, the entropy method, Pearson correlation coefficient, regression model, and coupling model to evaluate and analyze the level of ecological civilization construction [5,6]. Some scholars used the data envelopment analysis (DEA) game cross-efficiency model to construct an ecological civilization performance evaluation system [7]. In addition, several scholars have built a human-oriented indicator system of ecological civilization construction. Additionally, the development of ecological civilization can be studied using indicators such as the physiological balance, psychological imbalance, human–environment imbalance, human development, economic and environmental disharmony, and ecological civilization index [8,9]. Third, from the perspective of ecological civilization research, most scholars have studied the development of ecological civilization at the provincial, municipal, and rural levels [10,11,12,13]. Fourth, the results of existing studies have shown that the quality of economic development and nature conservation [14,15,16], clean energy investment, water supply for urban residents, foreign investment utilization, land resource protection, and population education quality [17] have significant effects on the level of ecological civilization construction. The results of these studies have shown that the industrial structure and marketization degree, government environmental risk awareness, government green action, residents’ happiness, corporate environmental responsibility, investment in culture and education, green travel and ecological construction, and other factors positively affect the performance of ecological civilization [7].

Based on the four above-mentioned aspects of research and progress, the significance of this study is as follows. Firstly, there have been a few systematic evaluations of the ecological civilization of watersheds. So far, the construction level of ecological civilization in subbasins in China has not been systematically evaluated. This study makes up for the lack of research on the evaluation of the construction level of ecological civilization from the perspective of watersheds. In the context of the gradual deepening of ecological environmental protection, the importance of the ecological protection of watersheds has become increasingly prominent. For example, the Yangtze and Yellow rivers, two mother river basins, have become China’s major strategic core. Therefore, it is necessary to systematically evaluate the ecological civilization of the whole basin. Secondly, in previous research, the absolute level of ecological civilization has been evaluated; however, the spatial characteristics of the cross-basin ecological civilization level have not been analyzed. Many rivers and lakes are distributed in China, and river basins are complex and diverse. Furthermore, the characteristics of river basins are very different. Therefore, the level of ecological civilization in river basins also has very notable spatial differences. One aim of this study was to explore the spatial evolution characteristics of the ecological civilization development level in China’s watersheds. Thirdly, the development level of watershed ecological civilization involves many aspects such as the environment, economy, politics, society, and culture. The internal characteristics that affect the overall level of ecological civilization in different watersheds also differ. One of the aims of this study was to explore the factors affecting the development level of watershed ecological civilization. Finally, the findings of this study provide theoretical references for optimizing policies regarding watershed ecological civilization construction.

This study is structured as follows: The second part contains the index system construction, data sources, and research process of Chinese watershed ecological civilization. In the third part, the evaluation results are analyzed using gap analysis between groups and panel regression analysis supported by GIS technology. In addition, the evolution of the temporal and spatial pattern and factors affecting the level of ecological civilization development in China are analyzed. The main conclusion and discussion are provided in the final section.

## 2. Methodology and Data Sources

### 2.1. Study Area

**Figure 1:**
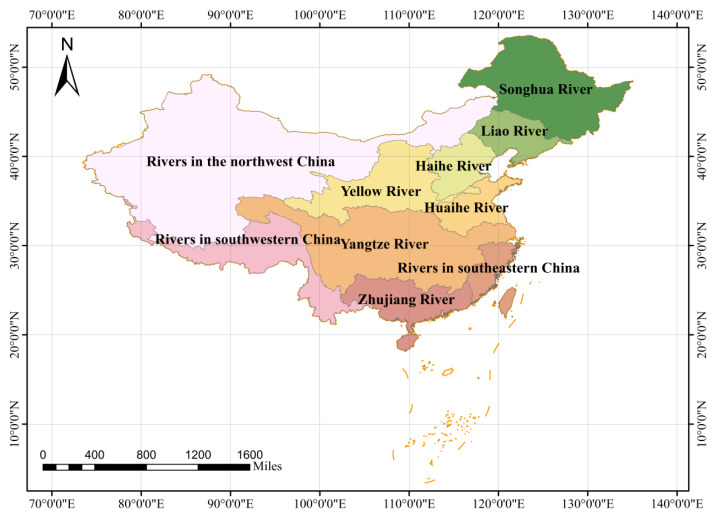
Study area.

According to the watershed system division standard of the Ministry of Water Resources of the People’s Republic of China [18], China can be divided into ten major river basins, namely Songhua River, Liao River, Haihe River, Yellow River, Huaihe River, Yangtze River, Zhujiang River, Rivers in southeastern China, Rivers in the northwestern China and Rivers in southwestern China. Therefore, we selected the above ten watersheds as the study area. Among them, Songhua River flows through 4 provinces (autonomous regions) including Heilongjiang Province, Jilin Province, Liaoning Province and the Inner Mongolia Autonomous Region; Liao River flows through 4 provinces (autonomous regions) including Hebei Province, Jilin Province, Liaoning Province and Inner Mongolia Autonomous Region; Haihe River flows through 8 provinces (cities and autonomous regions) including Beijing municipality, Tianjin municipality, Hebei Province, Henan Province, Shanxi Province, Shandong Province, Liaoning Province and the Inner Mongolia Autonomous Region; the Yellow River flows through 9 provinces (autonomous regions) including Shandong Province, Gansu Province, Henan Province, Shanxi Province, Qinghai Province, Shanxi Province, Sichuan Province, the Inner Mongolia Autonomous Region and Ningxia Hui Autonomous Region; Huaihe River flows through 5 provinces including Anhui Province, Henan Province, Hubei Province, Jiangsu Province and Shandong Province. The Yangtze River flows through 19 provinces (autonomous regions) including Shanghai Municipality, Chongqing Municipality, Anhui Province, Fujian Province, Gansu Province, Guangdong Province, Guizhou Province, Henan Province, Hubei Province, Hunan Province, Jiangsu Province, Jiangxi Province, Qinghai Province, Shanxi Province, Sichuan Province, Yunnan Province, Zhejiang Province, the Guangxi Zhuang Autonomous Region and Tibet Autonomous Region; The Zhujiang River flows through 9 provinces (autonomous regions) including Fujian Province, Guangdong Province, Guizhou Province, Hainan Province, Hunan Province, Jiangxi Province, Yunan Province, the Guangxi Zhuang Autonomous Region and Hong Kong Special Administrative Region; Rivers in southeastern China flow through 6 provinces including Anhui Province, Fujian Province, Guangdong Province, Jiangxi Province, Zhejiang Province and Taiwan Province; Rivers in northwestern China flow through 8 provinces (autonomous regions) including Gansu Province, Hebei Province, Qinghai Province, Shanxi Province, Inner Mongolia Autonomous Region, Ningxia Hui Autonomous Region, the Tibet Autonomous Region and Xinjiang Uygur Autonomous Region; Rivers in southwestern China flow through 5 provinces (autonomous regions) including Qinghai Province, Yunan Province, the Guangxi Zhuang Autonomous Region, Tibet Autonomous Region and Xinjiang Uygur Autonomous Region (Figure 1).

### 2.2. Evaluation Index System

#### 2.2.1. Principles for Building Indicator Systems

The core task of the construction of watershed ecological civilization is to realize the harmonious coexistence of humans and the watershed ecosystem. In time and space, the demand of human activities is the driving force, and the realization of watershed ecological civilization is the goal. Based on the input–output chain channels, scientific and technological procedures are organically combined to form an open system. Based on the interactions between various systems, an orderly and complex structure is finally formed to fulfill the functions of material circulation, energy flow, information transmission, and capital appreciation [19]. Considering the current status of the ecological environment of river basins, detailed evaluations at various levels are required, such as protecting the ecological environment, saving resources and energy, and optimizing land space, such that the evaluation system can be fully reflected in the whole process of building ecological civilization and so that the role of each subject in the construction of ecological civilization can be clarified. Therefore, the principles of integrity, guidance, and dynamic accessibility should be followed during the construction of the evaluation index system of watershed ecological civilization.

#### 2.2.2. Identification of Key Indicators

According to the basic principles of index construction, the spatial pattern of watershed development, resource and energy utilization efficiency, river ecosystem health, the human settlement environment, and the watershed ecological civilization system are fully considered. Referring to the idea of an ecological civilization evaluation index system as proposed by Dong Feng et al. [7], we developed an evaluation system for the construction of ecological civilization in watersheds that considers factors including the environment, economy, society, culture, and politics, in order to comprehensively and accurately reflect the level of ecological civilization construction of each river basin [20,21]. The established steps of the index system are as follows:

The first step is to confirm the five dimensions of evaluation. In 2012, the Chinese government proposed integrating ecological progress into the five-sphere integrated plan for building socialism with Chinese characteristics [22]. Therefore, this study comprehensively evaluates the development level of watershed ecological civilization from five dimensions: environment, economy, society, politics and culture. The second step is to select the corresponding evaluation indices according to the five dimensions. Based on the relevant studies on the evaluation of watershed ecological civilization and water ecological civilization, from the perspective of the sustainable development of the watershed, this study selects the indicators that are widely used in the existing literature from five dimensions as the alternative indicators for the evaluation of watershed ecological civilization. The third step is to discuss with relevant ecological civilization experts through a semi-structured interview and finally determine the watershed ecological civilization index system with 5 dimensions and 19 evaluation indicators., as shown in Table 1.

### 2.3. Research Methods

#### 2.3.1. Factor Analysis Method

The development level of watershed ecological civilization is comprehensive; a specific index to measure the level has not been established. Therefore, construction of a relevant index system is necessary. Since the relationships among multiple variables are complex and the values of the variables are not all in the same dimension, it is difficult to make direct comparisons. Therefore, factor analysis was adopted in this study to measure the ecological civilization of the watersheds. Factor analysis originated in the early 20th century and was first proposed by Charles Spearmen and Karl Pearson. Based on factor analysis, many variables can be reduced into several common factors containing most of the information to achieve dimensionality reduction, and the score of the common factor can be calculated by matching the corresponding weight to carry out an objective and scientific quantitative evaluation. Factor analysis through the study of multiple variables, can determine the potential behind a small number of driving factors, and can be used to describe the relationship between multiple variables. The few unobservable random variables are represented by F and ε, where F is called a common factor and ε is called a special factor. The factor analysis equation is as follows:

(1) $${\left[\begin{array}{c} X_{1}\\ X_{2}\\ \vdots \\ X_{P} \end{array}\right]}_{}=[\begin{array}{cccc} \begin{array}{c} a_{11}\\ a_{21}\\ \vdots \\ a_{p1} \end{array} & \begin{array}{c} a_{12}\\ a_{22}\\ \vdots \\ a_{p2} \end{array} & \begin{array}{c} \cdots \\ \cdots \\ \vdots \\ \cdots \end{array} & \begin{array}{c} a_{1m}\\ a_{2m}\\ \vdots \\ a_{pm} \end{array} \end{array}][\begin{array}{c} F_{1}\\ F_{2}\\ F_{3}\\ F_{m} \end{array}]+\left[\begin{array}{c} \varepsilon_{1}\\ \varepsilon_{2}\\ \vdots \\ \varepsilon_{P} \end{array}\right],$$

where $X_{1},X_{2},\ldots \ldots ,X_{P}$ are observable indices, $F_{1},F_{2}\ldots \ldots ,F_{m}$ is m common factors, and $a_{ij}$ is factor load; that is, the weight of the *i*th variable on the *j*th factor.

Additionally, where m<p. This relationship can be written as:

(2) X=AF+ε,

where A is the factor load matrix. F is the common factor matrix, ε is the special factor matrix. The model should meet the following assumptions:

(3) $$\{\begin{array}{c} E(F)=0\\ E(\varepsilon )=0\\ Var(F)=I\\ Var(\varepsilon )=diag(\sigma_{1}{}^{2},\cdots ,\sigma_{p})\\ Cov(F,\varepsilon )=0 \end{array}.$$

The establishment of the factor analysis model can be divided into the following steps:

Step 1: To reduce the order of magnitude and solve the problem of heteroscedasticity, range standardization was adopted to apply dimensionless standardization to the original data matrix. The index equation is as follows:

Positive indicators:

(4) $$x_{it}^{*}=\frac{x_{it}-\min(x_{i})}{\max(x_{i})-\min(x_{i})},$$

Negative indicators:

(5) $$x_{it}^{*}=\frac{\max(x_{i})-x_{it}}{\max(x_{i})-\min(x_{i})},$$

where $x_{it}$ refers to the original value of item i in the period t, $x_{it}^{*}$ refers to the standard value of the index in the investigation period, and $\max(x_{i})$ and $\min(x_{i})$ are the maximum and minimum value of item i, respectively.

In the second step, common factors were extracted. By calculating the eigenroots and eigenvectors of the covariance matrix, the number of common factors was determined based on the cumulative contribution rate of variance, and the factor loading matrix was obtained.

In the third step, factor rotation and factor explanation were carried out. The factor load matrix, which was directly obtained from the eigenroots and eigenvectors, may have a low differentiation of the factor load and weak explanatory power. Factor rotation can improve the differentiation of the same original variable in the load coefficient of each factor, such that the explanatory power of the key factor can be highlighted. Factor rotation methods mainly include orthogonal and oblique rotation. In this study, variance-maximizing rotation was used.

In the fourth step, the score of each factor was calculated. After determining the factor load matrix, factor score values of each sample were calculated using the least squares method.

After the above-mentioned factor analysis steps were completed, the proportion of the variance contribution rate of each common factor to the total variance contribution rate of the selected common factor $a_{i}$ and the score of each factor $F_{i}$ were used to calculate the factor comprehensive score: $F=\sum_{i=1}^{q}a_{i}F_{i}$, where F is the comprehensive score value of the factor as the evaluation index.

#### 2.3.2. Expert Scoring Method

Expert scoring method: The expert scoring method is a widely used method that uses the knowledge of experts to evaluate various indicators based on quantitative and qualitative analysis. Semi-structured interviews: The semi-structured interview is an intermediate method between structured interviews and unstructured interviews. It is more flexible than the structured interview, and the researchers will have key topics to study, as well as an interview guide. During the interview process, they can ask questions with flexibility based on these key points, and they can also discuss new questions. In this paper, on 24 November 2021, five researchers in the field of ecological civilization were selected as experts, including university professors and researchers in scientific research institutions. Additionally, semi-structured interviews were used to discuss and determine the evaluation index system of watershed ecological civilization.

#### 2.3.3. Group Gap Analysis

In this study, the standard deviation and coefficient of variation were used to describe the degree of deviation of the ecological civilization level difference in Chinese river basins. The following equations were used:

(6) $$\delta =\sqrt{\sum_{i=1}^{N}{\left(y_{i}-\bar{y}\right)}^{2}/N},$$

(7) $$cv=\delta /\bar{y},$$

where $y_{i}$ represents the ecological civilization level of a certain river basin (*i* = 1, 2, 3,…, *N*; *N* is the number of river basins) and $\bar{y}$ is the average ecological civilization level of the river basins.

#### 2.3.4. Panel Regression Analyses Method

The panel model combines the common advantages of the time series and cross-sectional data models. In this study, the panel model was adopted to test the explanatory ability of these factors with respect to the development level of watershed ecological civilization. The model was set as follows:

(8) $$Y_{it}=\beta_{0}+\beta_{1}\ln X_{1it}+\beta_{2}\ln X_{2it}+\beta_{3}\ln X_{3it}+\beta_{4}\ln X_{4it}+\beta_{5}\ln X_{5it}+\varepsilon^{},$$

where i is the section, t is the time, $\beta_{j}$ (j = 0,1,2,3,4,5) is the regression coefficient, $Y_{it}$ is the comprehensive score of the ecological civilization development level of watershed i in year t, $X_{1it}$ is the FDI level of watershed i in year t, $X_{2it}$ is the urbanization rate level of watershed i in year t, $X_{3it}$ represents the forest coverage rate watershed i in year t, $X_{4it}$ represents the level of education of watershed i in year t, $X_{5it}$ represents the level of financial autonomy of watershed i in year t, and ε is the random disturbance term.

#### 2.3.5. The Framework of Study

**Figure 2:**
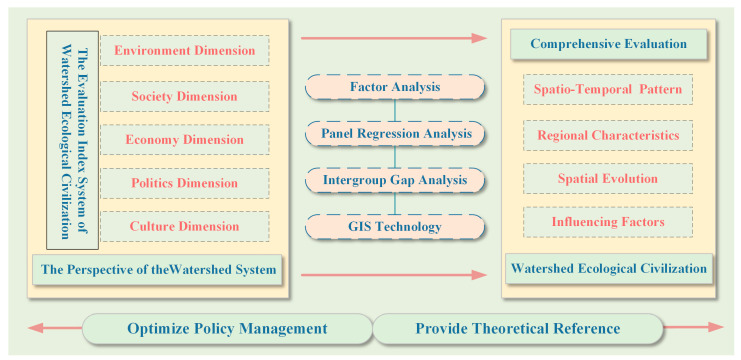
The framework of study.

In this study, provincial panel data were used to construct the evaluation index system of watershed ecological civilization covering five dimensions: environment, society, economy, politics, and culture. Factor analysis was used to evaluate the construction level of ecological civilization in China’s top ten basins and to determine the development status of ecological civilization in China’s top ten basins from 2004 to 2018. Based on the regression analysis of panel data, important factors affecting the construction level of ecological civilization in China’s basins were further analyzed and explored to provide a decision making basis and empirical evidence for the construction of watershed ecological civilization to promote the harmonious coexistence of people and the natural environment (Figure 2).

### 2.4. Data Sources

Due to the unavailability of relevant data on the basin boundaries, we selected the inter-provincial panel data of 2004–2018 as the sample data, based on the scientific nature and availability of the data. The panel data of 30 provinces were obtained from the China Statistical Yearbook, China Industrial Statistical Yearbook, China Science and Technology Statistical Yearbook, China High-tech Industry Statistical Yearbook, China Regional Economic Statistical Yearbook, China Energy Statistical Yearbook, China Water Conservancy Statistical Yearbook, China Environment Statistical Yearbook, and statistical yearbooks and statistical bulletins of provinces (cities, districts). All the above databases are from China’s economic and social big data research platform (https://data.cnki.net/ accessed on 27 November 2021).

## 3. Results

### 3.1. Comprehensive Assessment of Watershed Ecological Civilization

Based on the factor analysis method, IBM SPSS Statistics for Windows (Version 20.0. IBM Corp.: Armonk, NY, USA) was used to analyze the ecological civilization index system of all watersheds in China.

#### 3.1.1. Feasibility Test

The feasibility of the factor analysis of the index data was tested. The test results are shown in Table 2. The Kaiser–Meyer–Olkin (KMO) test value of the sample was 0.669, indicating a high degree of overlap of information among the indicators. The approximate chi-square value of Bartlett’s spherical test was 4380.38 and the significance probability (Sig) was 0.000 when there were 171 degrees of freedom (df). This shows that the independence hypothesis for each index was not tenable. Therefore, the index data were suitable for factor analysis.

#### 3.1.2. Variance Contribution Rate

Maximum likelihood estimation was adopted for factor estimation. The results are shown in Table 3. The cumulative variance contribution of seven factors reached 76%, and information about 19 indicators could be provided. Therefore, it was reasonable to select seven factors for the follow-up analysis.

#### 3.1.3. Factor Load

The factor load coefficient matrix of each ecological civilization evaluation index for the seven factors was estimated based on the maximum likelihood, and factor rotation was carried out according to the principle of variance maximization. The results are shown in Table 4.

#### 3.1.4. Comprehensive Factor Score

Based on the variance contribution rate and factor load coefficient of each index, the comprehensive factor score for each province was calculated using the following equation:

(9) $$ECrp_{j}{}_{t}=\frac{\left(V_{t,1}\times F_{t,1}+V_{t,2}\times F_{t,2}+V_{t,3}\times F_{t,3}+V_{t,4}\times F_{t,4}+V_{t,5}\times F_{t,5}+V_{t,6}\times F_{t,6}+V_{t,7}\times F_{t,7}\right)}{\sum_{n=1}^{7}V_{tn}},$$

where $F_{t,n}$ represents the score of the nth factor in year t for a given province and $V_{t,n}$ represents the variance contribution rate of the nth factor in year t.

#### 3.1.5. Calculation of the Ecological Civilization Score of the River Basin

The ecological civilization score for each province in a given river basin was obtained through factor analysis. Finally, the ecological civilization score for each river basin was calculated by the following equation:

(10) $$ECrb_{i}{}_{t}=ECrp_{j}{}_{t}\cdot \frac{S_{ij}}{S_{i}},$$

where $ECrb_{i}{}_{t}$ is the ecological civilization score of river basin i in year t, $ECrp_{j}{}_{t}$ is the ecological civilization score of province j in year t, $S_{ij}$ is the total area of province j in river basin i, and $S_{i}$ is the total area of river basin i.

To facilitate the analysis, we finally selected the scores for the ecological civilization development level in 2004, 2008, 2013, and 2018 to analyze the development level of ecological civilization in China’s watersheds from 2004 to 2018, as shown in Table 5. Table 5 shows that in terms of the comprehensive score ranking of ecological civilization, the Haihe River Basin and Liaohe River Basin held the top two scores of ecological civilization in the ten basins of China in 2004, 2008, 2013, and 2018, and the southwestern rivers ranked in the last places. In terms of specific subbasins, compared with 2004, the scores of the ecological civilization development level in the southeastern river basins decreased the most in 2018, from sixth to ninth place, and the ranking of the Huaihe River Basin decreased by two places. The rankings of other basins changed little, indicating that the overall level of ecological civilization development in Chinese basins is relatively stable. However, compared with 2004, the level of ecological civilization construction in major river basins in 2018 had markedly improved, but the level of ecological civilization construction in the southwestern river basins remained low.

### 3.2. Analysis of the Temporal and Spatial Evolution of Watershed Ecological Civilization

#### 3.2.1. Spatial Characteristics

**Figure 3:**
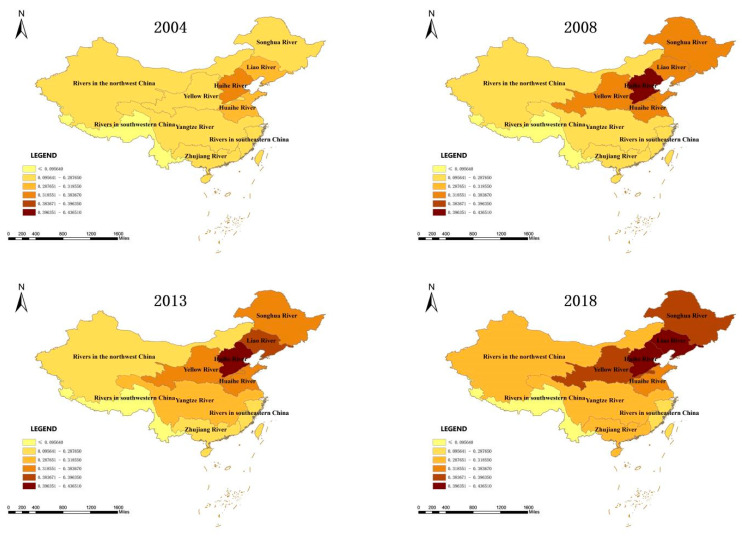

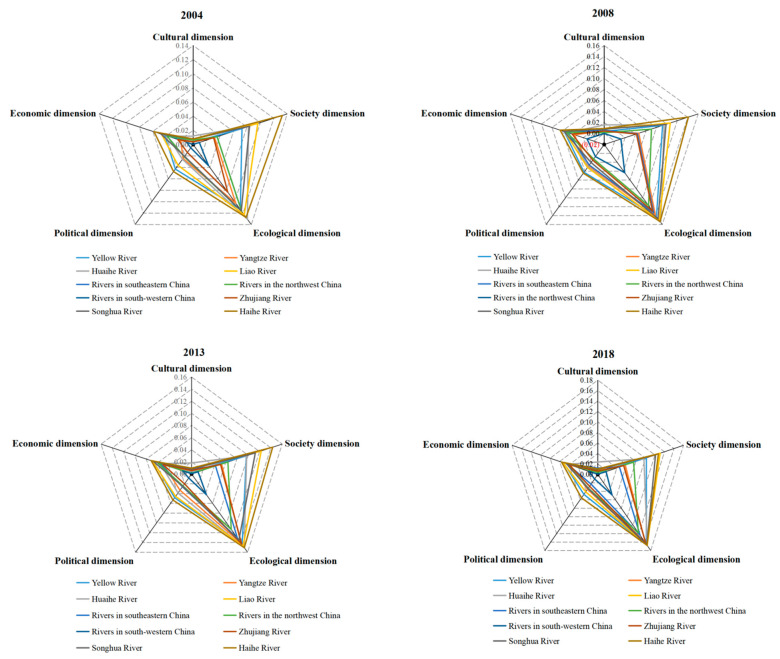
Spatial distribution of the ecological civilization level in Chinese river basins in 2004, 2009, 2014, and 2018.

To better present regional differences in the level of ecological civilization in river basins, the two-dimensional spatial and temporal characteristics were highlighted to explore the agglomeration differences in the evolution of ecological civilization in various river basins over time. In this study, ArcGIS10.2 software (Release 10.2. Environmental Systems Research Institute: Redlands, CA, USA) was used to draw spatial distribution maps of the ecological civilization in various dimensions of river basins in 2004, 2008, 2013, and 2018 (Figure 3).

Figure 3 shows that the level of ecological civilization in China’s watersheds continuously improved over time. In 2018, the overall ecological civilization level of the river basins increased by 42.4% compared with that of 2004, which is reflected in the continuous spread from the Haihe River Basin to the Liaohe, Yellow, Songhua, and other peripheral river basins. The improvements of the ecological civilization level in the Pearl and Yangtze River regions were the largest. The level increased by 2 and 1.76 times, respectively.

In recent years, great leaps have been made in the economic development of the Yangtze and Pearl River regions, and great progress has been made in the ecological environment management of the basins, which has greatly improved the level of ecological civilization. In terms of the five dimensions, the ecological civilization level of the ecological dimension is the highest, followed by the social and economic dimensions. The ecological civilization level of the political and cultural dimensions is relatively weak. However, all dimensions of ecological civilization show a notable upward trend. The ecological civilization levels of the economic, cultural, and political–ecological–social dimensions in 2018 increased by 69.3%, 45.8%, and 30–45%, respectively, compared with those in 2004.

From the point of view of typical river basins, the ecological civilization level of Chinese river basins is gradually increasing from southwest to east. The level of ecological civilization is the highest in the Haihe Region, followed by the Liaohe, Yellow River, Huaihe River, and Songhua River regions. The level is the lowest in the southwestern river region. However, there are some differences with respect to the five dimensions. The ecological civilization level of the Huaihe River Region ranks first in terms of the cultural and ecological dimensions, and its performance is the most outstanding. The Haihe, Yellow River, and Liaohe districts rank first with respect to the level of ecological civilization in the political dimension, possibly because Beijing, as the political center of China, scores highly in this region. In 2018, the ecological civilization level of the Yangtze River Region in the economic dimension was increased by 3.12 times compared with that in 2004, and the ecological civilization level of the Pearl River Region in the economic, political, and ecological dimensions increased by 2.63, 1.99, and 1.96 times, respectively. In 2018, the ecological civilization level of the river basins increased by 2.20 times compared with that in 2004. The northwestern river basin has the largest room for improvement concerning the ecological civilization level, which increased by 2.11 times compared with 2004.

#### 3.2.2. Evolution of the Temporal Pattern

**Figure 4:**
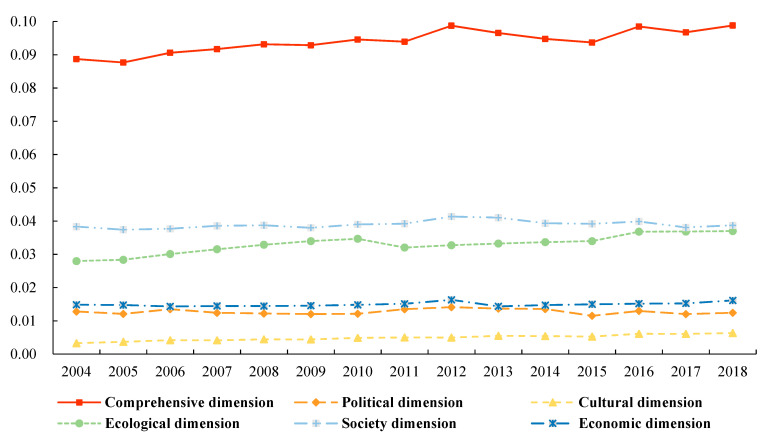
Variation in the standard deviation of ecological civilization in Chinese watersheds.

**Figure 5:**
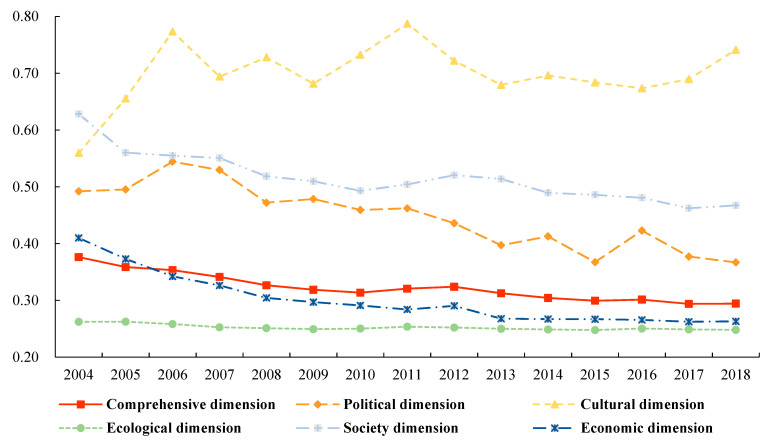
Variation in the coefficient of ecological civilization in Chinese watersheds.

The standard deviation and coefficient of variation in the ecological civilization of China’s watersheds were calculated using Equations (6) and (7). The results are plotted in Figure 4 and Figure 5, respectively. Based on the variation in the standard deviation of each dimension, the standard deviation can be ranked as follows: comprehensive dimension > social dimension > ecological dimension > economic dimension > political dimension > cultural dimension. Based on the variation in the coefficient of variation in each dimension, the coefficient of variation can be ranked as follows: cultural dimension > social dimension > political dimension > comprehensive dimension > economic dimension > ecological dimension. From the perspective of the time axis variation trend, the variation coefficient of ecological civilization of the cultural dimension fluctuates with time, indicating that the dispersion degree of ecological civilization of the cultural dimension is expanding. The coefficients of the variation in the economic, political, and social dimensions have gradually declined over time, indicating that the degree of clustering of watershed ecological civilization scores in the comprehensive, economic, political, and social dimensions is increasing. However, the decline curves are not the same. The coefficient of variation in the economic dimension has dropped the fastest; the value of the coefficient of variation in 2018 is 64.2% of that in 2004. The decreasing rates of the political and social peacekeeping dimensions gradually decreased (74.4%, 74.6%, and 78.2%), indicating that the degree of agglomeration of the ecological civilization level of the economic dimension is the most notable and the ecological dimension is the most stable. The coefficient of variation in the ecological dimension only decreased by 5.4% from 2004 to 2018, indicating that the degree of dispersion of the ecological civilization of the watershed did not change considerably.

### 3.3. Analysis of Factors Affecting the Development of Ecological Civilization in Chinese River Basins

#### 3.3.1. Variable Selection

In this section, the influencing factors of the development level of ecological civilization in ten river basins in China are studied. The dependent variable is expressed by the comprehensive factor (Y) of the development level of ecological civilization obtained by factor analysis. The indices of factors affecting the development level of ecological civilization in the watersheds were determined based on the existing literature. The independent variables are as follows:

(1) Degree of opening to the outside world: In this study, foreign direct investment (FDI) was used to represent the degree of opening to the outside world. The results of previous studies showed that several scholars believe that enterprises in developed countries will transfer their high-polluting and energy-consuming production activities to developing countries with lower environmental pollution costs, which will damage the environment and ecology of developing countries receiving these enterprises. However, other scholars believe that FDI has a technology spillover effect and can bring advanced green production technology to the host country, thus improving its environmental quality [23,24,25]. Therefore, FDI may either improve or destroy the ecological environment of the host country, and the results remain uncertain.
(2) Urban development level: In this study, the urbanization rate; that is, the proportion of the population that is urban, was used to represent the level of urban development. On one hand, a high level of urbanization can promote economic development and social progress; on the other hand, local resource consumption and pollution discharge will increase due to the agglomeration of the urban population, leading to excessive pressure on the ecological environment. Therefore, the effect of the urban development level on the development level of ecological civilization in watersheds remains uncertain.
(3) Forest coverage level: The forest coverage rate was selected as the index of the forest coverage level. Forests play a key role in improving ecological vitality, and the forest coverage rate and ecological vitality are markedly positively correlated. Ecological construction is the natural foundation of ecological civilization construction and improving ecological vitality promotes ecological civilization construction [5]. Therefore, the forest cover level is expected to have a positive effect on the development level of ecological civilization in watersheds.
(4) Education level: Education level is expressed as the average number of students in schools of all levels per 100,000 people. The improvement in the regional education level is conducive to shaping a good concept of ecological civilization and promoting its construction. The education level has a significant positive effect on the development of local and surrounding ecological civilization [15]. Therefore, it is expected that the level of education will be beneficial to the construction of ecological civilization in the watershed.
(5) Fiscal decentralization: The results of previous studies showed that government investment is one of the factors affecting the development level of ecological civilization [7]. In this study, fiscal autonomy was expressed by dividing the fiscal revenue in the regional budget by the total fiscal expenditure in the budget. On one hand, a government paying too much attention to economic growth while ignoring environmental problems will lead to an extensive development mode of ecological environment destruction. On the other hand, local fiscal expenditures tend to favor public services and external welfare improvements, which favor ecological improvements with uncertain outcomes.

#### 3.3.2. Empirical Test and Regression Analysis

(1) Panel inspection

To test whether the data results are affected by the time series and whether there is a long-term stationary relationship between variables, panel unit root and cointegration tests were carried out. To ensure the validity of the test results, the Levin–Lin–Chu (LLC) and Augmented Dickey–Fuller (ADF) methods were used for the panel unit root test. Table 6 shows that the variables Y, LnX_1_, LnX_2_, LnX_3_, LnX_4_, and LnX_5_ passed the 1% significance level test. The above-mentioned six variables thus meet the requirements of stationary time series. The Predoni method was adopted for the cointegration test. The test results in Table 7 indicate a long-term stable cointegration relationship among variables. Thus, the next step of the panel data estimation can be carried out.

(2) Model testing

The static panel model, which must be determined by the F- and Hausman tests, was adopted in this study. Table 8 shows that the model failed both the Hausman test and panel regression with random effects regression models. As can be seen from Table 9, four independent variables in the uncorrected random effects model passed the significance test. The coefficients of two variables passed the 1% significance level test, again indicating the applicability of the random effects model.

Since parameter T used in this study was greater than N (T = 15, N = 10), the data in this study were long-panel data. Considering that the disturbance term may have heteroscedasticity and autocorrelation, a correlation test under the random effect model was carried out. The test results in Table 8 show that the null hypothesis of “homoscedasticity” was strongly rejected and intergroup heteroscedasticity existed under the modified Wald test. Wooldridge’s test strongly rejected the null hypothesis that there was no first-order intra-group autocorrelation, and it considered that there was intragroup autocorrelation. The Lagrange multiplier (LM) test strongly rejected the null hypothesis of “no contemporaneous correlation” and considered the existence of a contemporaneous correlation. Based on these test results, the random effect model was for the regression and the comprehensive feasible generalized least squares method (FGLS) was used to deal with the problems of heteroscedasticity, autocorrelation, and contemporaneous correlation between groups.

(3) Analysis of the model results: FGLS estimation

Based on the regression model results in Table 9, the comprehensive RANDOM effect model of FGLS panel data was adopted after the modification of intragroup heteroscedasticity, autocorrelation, and contemporaneous correlation. Four of the five explanatory variables passed the significance level test, and the model results were ideal. Therefore, the factors affecting the development level of ecological civilization in the watersheds can be discussed based on the comprehensive FGLS panel data stochastic effect model.

① Degree of openness

The model regression results in Table 9 show that the openness degree of the FDI in China’s top ten river basins during 2004–2018 had a positive effect on the overall ecological civilization of the basin. The T value of FDI was 13.07, which passed the 1% significance level test. The results of this study and previous work indicate that foreign investment will lead to high pollution and energy transfer in the host country, which has a negative effect on the level of development of ecological civilization. From another point of view, the technology spillover effect of FDI may bring advanced green production technology to the host country and improve the ecological environment quality, thus promoting the development level of ecological civilization in the river basins. Foreign investment will bring highly polluting and energy-consuming enterprises to the host country. However, the host country’s environmental regulation intensity will increase with economic development, forcing foreign investments with advanced green production technology upgrades to meet the needs of environmental regulation. This technology spillover effect will lead to the upgrading of green technology in the surrounding industries of the host country. Finally, the development level of ecological civilization of the host country watershed is promoted.

② Level of urban development

The model regression results in Table 9 show that the urban development level of the ten river basins in China from 2004 to 2018; that is, the improvement of the urbanization rate, inhibited the development level of ecological civilization in the river basins. The T value of the urbanization rate was −12.33, passing the 1% significance level test. An improvement in the urban development level generally refers to significant increases in the regional population and industrial concentration. If the level exceeds the optimal carrying capacity of the ecological environment, the ecological environment will be damaged, which will have a negative impact on the development level of ecological civilization in the watershed. Therefore, the level of urban development restrains the level of ecological civilization development.

③ Level of forest cover

The model regression results in Table 9 show that the forest cover level of the ten river basins in China from 2004 to 2018; that is, the improvement of the forest coverage rate, had a positive effect on the development level of ecological civilization in the basins. The T value of the forest coverage rate was 7.83, passing the 1% significance level test. Forests can improve the local ecological vitality, and the ecological vitality promotes the construction of ecological civilization. The research results of this model verify our hypothesis that the forest cover level notably promotes the construction of ecological civilization in the basin.

④ Education level

The model regression results in Table 9 show that the education level in the ten river basins of China from 2004 to 2018; that is, the improvement in the average number of school students at all levels per 100,000 people, had a positive effect on the development level of ecological civilization in the river basins. The T value of the average number of students in schools at all levels per 100,000 people was 1.91, passing the 10% significance level test. The improvement of the education level will help to improve people’s awareness of ecological environmental protection and form the concept of active construction of ecological civilization in society to promote the construction of ecological civilization in the river basins.

⑤ Fiscal decentralization

The model regression results in Table 9 show that the level of local fiscal autonomy; that is, the fiscal decentralization index, restrained the development level of ecological civilization of the ten river basins in China from 2004 to 2018. The T value of the fiscal decentralization index was −6.44, which passed the 1% significance level test. The results of this model support the view that the government pays too much attention to economic growth and ignores environmental problems. The extensive development mode leads to the destruction of the ecological environment, which is not conducive to the development of ecological civilization in the basins. Therefore, the higher the degree of local financial autonomy, the more unfavorable it is to the development of ecological civilization in the watersheds.

## 4. Discussion

Based on the interprovincial panel data of China’s top ten river basins flowing through provinces from 2004 to 2018, a comprehensive evaluation index system of river basin ecological civilization was constructed in this study. The factor analysis method was used to evaluate the level of development of river basin ecological civilization in China, and the spatiotemporal evolution was analyzed with GIS technology. Furthermore, the panel regression model was used to explore the factors affecting the development level of ecological civilization in Chinese watersheds.

First, with respect to the spatiotemporal evolution and evolutionary characteristics of the time series pattern, Haihe River Basin is the location of Beijing, the capital of China, and is the political, economic and cultural center of China. It has high scores of ecological civilization in political, economic and cultural dimensions. Therefore, the development of ecological civilization in the Haihe River Basin is the best. However, due to China’s economic development, China’s economic center has been moving southward, including several enterprises with high pollution and high emissions. The north has always been the key area of environmental governance in China, leading to a higher development level of ecological civilization in the northern basin. The economy has developed rapidly in the Yangtze and Pearl River regions in the past decade; this explains the fastest improvement in the economic dimension of the ecological civilization development level.

Second, from the perspective of the influencing factors on the development level of ecological civilization in China’s river basins, the reasons are as follows: (1) foreign investments have a technology spillover effect, which can bring advanced green production technology to the local area, improve the quality of the ecological environment, and thus promote the development of ecological civilization in the basin; (2) forests can improve local ecological vitality, which in turn improves the level of ecological dimension in the construction of ecological civilization; (3) the improvement of the education level promotes people’s awareness of ecological environmental protection, and it is easy to societally form the concept of actively building ecological civilization so as to promote the level of watershed ecological civilization construction; (4) improvement in the urban development level generally reflects significant increases in the regional population and industrial concentration; if the level exceeds the optimal carrying capacity of the ecological environment, the ecological environment will be damaged, which will have a negative effect on the development level of the ecological civilization of the basin; and (5) local governments place too much emphasis on economic growth while ignoring environmental problems, resulting in extensive development and the destruction of the ecological environment, which is not conducive to the development of ecological civilization in the river basin.

Third, this study objectively evaluates the development status of ecological civilization and its influencing factors in Chinese watersheds from the perspective of the whole watershed and provides new ideas for the evaluation of ecological civilization construction, as well as decision support for the government in the construction of an ecological civilization. Although this work has practical significance, several limitations remain. In this study, the provincial panel data of each watershed were used, and the ecological civilization of the watershed was evaluated using a weighted portion of the whole watershed area, which may have affected the accuracy of the results to a certain extent. Data from cities or counties should be used for further research. Furthermore, the evaluation indicators used in this study were selected based on previous research and, thus, are subjective. The factors affecting the development level of ecological civilization in the basin must be further explored in subsequent studies.

## 5. Conclusions

The results from this paper support the following conclusions.

(1) The level of ecological civilization in Chinese watersheds has continuously developed for the better.
(2) The development level of ecological civilization in Chinese watersheds shows a tendency to gradually spread from the Haihe River basin to the peripheral watersheds such as the Liaohe River area, the Yellow River area, and the Songhua River area, and it tends to gradually increase from southwest to east.
(3) The higher degree of openness, forest coverage, and education level can promote the development level of ecological civilization in the basins.
(4) The higher level of urban development and financial autonomy will inhibit the development level of ecological civilization in the basins.

## Figures and Tables

**Table 1:** Index system of watershed ecological civilization.

First Grade Index	Second Index	Attribute	Unit	Explanation
Economic dimension	Water consumption of 10,000-yuan industrial added value	Negative	m^3^/million	Total water consumption of industrial enterprises above scale/added value of industrial enterprises above scale
	Average water consumption per hectare of farmland irrigation	Positive	million m^3^/ha	Irrigation water consumption/crop planting area
	Proportion of water-saving irrigation area	Positive	%	Water-saving irrigation area/irrigation area
	Water savings per unit industrial enterprise	Positive	10,000 m^3^/unit	Urban industrial water conservation/number of industrial enterprises above scale
Society dimension	Proportion of the groundwater supply	Positive	%	supply amount of groundwater/total water supply quantity
	Treatment rate of domestic sewage	Positive	%	Treatment rate of domestic sewage
	Ecological water supplement per capita	Positive	m^3^/person	Ecological water supplement/total population
	Reuse of actual water per unit	Positive	%	Urban industrial water reuse/actual urban planned water consumption
Ecological dimension	Chemical oxygen demand emissions per million yuan GDP	Negative	ton/yuan	Chemical oxygen demand emissions/GDP
	Ammonia nitrogen emissions per million yuan GDP	Negative	ton/yuan	Ammonia nitrogen emissions/GDP
	10,000 yuan GDP wastewater discharge	Negative	ton/yuan	Wastewater emissions/GDP
	Unit added value of industry water conservation and ecological water conservancy investment	Positive	%	Investment in water conservation and ecological water conservancy/value added of industrial enterprises above scale
Cultural dimension	Number of national water-saving cities	Positive	law of individual variance	Number of national water-saving cities
	National Water Conservancy Scenic Area	Positive	law of individual variance	National Water Conservancy Scenic Area
	Proportion of wetland area in the area of jurisdiction	Positive	%	Wetland area/urban area
Political dimension	Water resources utilization ratio	Positive	%	Water resources utilization ratio
	Proportion of environmental pollution control investment in GDP	Positive	%	Environmental pollution control investment/GDP
	New proportion of soil erosion control area per unit area of jurisdiction	Positive	%	Increased soil erosion control area/jurisdiction area
	Total investment of 10,000 yuan GDP central water conservancy construction plan	Positive	%	Central Water Conservancy Project Investment Total/GDP

**Table 2:** Kaiser–Meyer–Olkin (KMO) and Bartlett’s tests.

	Sample Sufficient Degree of KMO Metrics	0.669
Bartlett’s spherical test	Approximate chi-square	4380.380
	df	171
	Sigma	0.000

**Table 3:** Factor variance contribution rates.

Factor	F1	F2	F3	F4	F5	F6	F7
Variance	4.309	2.462	2.309	1.856	1.397	1.069	1.025
Variance contribution rate	0.23	0.13	0.12	0.10	0.07	0.06	0.05
Cumulative contribution rate	0.23	0.36	0.48	0.58	0.65	0.71	0.76

**Table 4:** Factor scoring coefficient matrix.

First Grade Index	Second Index	Factor						
		F1	F2	F3	F4	F5	F6	F7
Economic dimension	Water consumption of 10,000-yuan industrial added value	0.264	0.123	−0.163	0.166	−0.015	−0.297	−0.223
	Average water consumption per hectare of farmland irrigation	0.025	−0.347	−0.004	0.191	0.044	0.066	0.095
	Proportion of water-saving irrigation area	0.065	0.130	0.030	−0.018	0.042	0.089	−0.013
	Water saving per unit industrial enterprise	−0.027	0.018	−0.005	0.017	−0.022	0.065	0.113
Society dimension	Proportion of groundwater supply	−0.039	0.205	0.007	0.080	0.554	0.050	0.247
	Treatment rate of domestic sewage	0.176	0.050	−0.037	−0.025	−0.106	0.117	−0.270
	Ecological water supplement per capita	0.000	0.304	−0.005	−0.125	0.064	−0.058	0.142
	Reuse of actual water per unit	−0.018	0.002	−0.076	−0.052	−0.100	0.828	−0.028
Ecological dimension	Chemical oxygen demand emissions per million yuan GDP	0.462	−0.263	−0.001	−0.007	−0.001	−0.209	0.397
	Ammonia nitrogen emissions per million yuan GDP	0.202	0.036	−0.031	−0.042	0.031	−0.012	−0.031
	10,000 yuan GDP wastewater discharge	−0.088	0.033	0.023	−0.070	0.025	0.177	0.356
	Added value of unit industry water conservation and ecological water conservancy investment	−0.058	0.121	0.024	0.058	−0.495	0.137	0.318
Cultural dimension	Number of national water−saving cities	0.053	0.033	−0.028	0.033	0.033	−0.005	−0.073
	National Water Conservancy Scenic Area	0.020	−0.022	0.011	0.012	−0.143	0.026	0.012
	Proportion of wetland area in the area of the jurisdiction	0.095	−0.068	0.816	−0.601	0.172	0.227	−0.116
Political dimension	Water resources utilization ratio	0.017	0.177	0.076	0.105	0.029	−0.019	−0.216
	Proportion of environmental pollution control investment in GDP	−0.055	0.159	0.060	0.069	0.010	0.096	−0.046
	New proportion of soil erosion control area per unit area of the jurisdiction	−0.011	−0.015	0.261	0.436	−0.087	0.038	−0.102
	Total investment of 10,000 yuan GDP in central water conservancy construction plans	−0.166	−0.012	0.234	0.529	−0.089	−0.250	0.200

**Table 5:** Comprehensive scores and rankings of ecological civilization in ten river basins in China.

Watershed	2004		2008		2013		2018	
	Score	Rank	Score	Rank	Score	Rank	Score	Rank
Haihe River	0.37321	1	0.41250	1	0.42729	1	0.43651	1
Liao River	0.31006	2	0.35217	2	0.38562	2	0.42169	2
Huaihe River	0.28910	3	0.34416	3	0.36687	3	0.38367	5
Songhua River	0.27613	4	0.33082	5	0.34850	5	0.39635	3
Yellow River	0.27475	5	0.33644	4	0.36588	4	0.39112	4
Rivers in southeastern China	0.21886	6	0.25896	6	0.27296	8	0.28765	9
Rivers in the northwestern China	0.21886	7	0.25421	8	0.27193	9	0.31364	7
Yangtze River	0.18095	8	0.25428	7	0.28859	6	0.31855	6
Zhujiang River	0.15451	9	0.23275	9	0.28098	7	0.31233	8
Rivers in southwestern China	0.06182	10	0.07575	10	0.08089	10	0.09564	10

**Table 6:** Unit root test.

Variable	LLC	ADF	
Y	−4.2986 ***	P61.7565 ***	Z-4.9235 ***
		L *-5.1794 ***	Pm6.6023 ***
LnX_1_	−6.8159 ***	P47.4533 ***	Z-3.7415 ***
		L *-3.8270 ***	Pm4.3407 ***
LnX_2_	−6.8467 ***	P50.9162 ***	Z-4.2171 ***
		L *-4.2427 ***	Pm4.8883 ***
LnX_3_	−17.0669 ***	P42.7412 ***	Z-3.4789 ***
		L *-3.4288 ***	Pm3.5957 ***
LnX_4_	−2.2495 **	P48.8862 ***	Z-3.5860 ***
		L *-3.6286 ***	Pm4.5673 ***
LnX_5_	−13.4728 ***	P49.5265 ***	Z-3.9259 ***
		L *-4.0159 ***	Pm4.6685 ***

*, **, and *** are significant at the 10%, 5%, and 1% levels, respectively.

**Table 7:** Cointegration test.

Statistic	Test
Modified Phillips–Perron t	3.4924 ***
Phillips–Perron t	−3.5673 ***
Augmented Dickey–Fuller t	−4.1899 ***

*, **, and *** are significant at the 10%, 5%, and 1% levels, respectively.

**Table 8:** Model test results.

Project	Mixed OLS	Fixed Effects	Random Effects	Generalized Least Squares
F-test			80.56 ***	
Hausman test		10.22		
Modified Wald test for groupwiseheteroscedasticity			166.57 ***	
Wooldridge test forautocorrelation in panel data			10.797 ***	
LM simultaneous correlation test between groups			166.712 ***	

*, **, and *** are significant at the 10%, 5%, and 1% levels, respectively.

**Table 9:** Regression results of the models.

	Mixed OLS Model	Random Effect Model	Fixed Effect Model	Comprehensive FGLS
Explanatory variables	Model 1	Model 2	Model 3	Model 4
LnX_1_	0.0174 ***	0.0058 *	0.0049	0.0091 ***
	(4.01)	(1.68)	(1.42)	(13.07)
LnX_2_	0.2729 ***	0.2233 ***	0.1899 ***	−0.1212 ***
	(17.14)	(13.82)	(9.96)	(−12.33)
LnX_3_	−0.0667 ***	−0.0154	−0.0009	0.0198 ***
	(−9.34)	(−1.46)	(−0.08)	(7.83)
LnX_4_	−0.1051 ***	−0.0730 **	−0.1094 ***	0.0167 *
	(−6.80)	(−2.38)	(−3.23)	(1.91)
LnX_5_	−0.0195	−0.0701 ***	−0.0997 ***	−0.0338 ***
	(−0.87)	(−3.72)	(−4.8)	(−6.44)
R-squared	0.8370	0.7451	0.7772	-
F-statistic	-	-	80.56 ***	-
Wald	-	465.79 ***	-	91,638.94 ***
Observed Value	150	150	150	150

*, **, and *** are significant at the 10%, 5%, and 1% levels, respectively.

## Data Availability

The data presented in this study are available on request from the corresponding author.
